# The Hospital. Nursing Section

**Published:** 1904-08-06

**Authors:** 


### The Hospital

### Nursing Section.

**Contributions for this Section of "The Hospital" should be addressed to the Editor, "The Hospital" Nursing Section, 28 & 29 Southampton Street, Strand, London, W.C.**

No. 932.—Vol. XXXVI. *SATURDAY, AUGUST* 6, 1904.

### Notes on News from the Nursing World.

### PHOTOGRAPHS OF THE RECEPTION AT BUCKINGHAM PALACE.

In reply to the numerous inquiries which we have received from members of the Royal National Pension Fund for Nurses who were present at the reception by the Queen at Buckingham Palace, we are glad to be able to announce that four distinct very successful whole-plate photographs were taken of the ceremony on July 22. Copies of these, handsomely mounted, may be obtained, post free, 1s. 6d. each, from the Manager of the Scientific Press, 28 and 29 Southampton Street, Strand, London, W.C.

### "THE HOSPITAL" CONVALESCENT FUND.

On another page will be found the acknowledgment of a sum of 10s. to The Hospital Convalescent Fund, per the *Southern Daily Mail*. The editor writes :—"In a competition in the columns of our recent issues a prize of 10s. was awarded to Miss Maud Ruel, and we have received a request from her to forward the amount to you for the benefit of the Convalescent Fund." This contribution embodies such a kindly and generous thought that we feel sure the idea will appeal to others who will be only too glad, now it has been suggested to them to take advantage of such unexpected opportunities of benefiting the fund without any deprivation to themselves by the depletion of their often slender purse.

### THE QUEEN AND THE MIDWIVES ACT.

Her Majesty the Queen has shown the practical interest she takes in the working of the Midwives Act by graciously consenting to become patron of the Association for Promoting the Training and Supply of Midwives. As our readers are aware, the object of the Association is to organise and assist the training of women to act as midwives among the poor, and thus to meet the requirements of the Act. With the Queen as patron, the Association cannot fail to increase its influence and to attract the support which it merits.

### RYAN v. THE CROWN.

A remarkable case was heard in the King's Bench Division on Monday. The plaintiff was Miss Elizabeth Ryan, formerly of the Army Nursing Service. She joined the service in 1885, and in June 1901 she was promoted to the position of nursing superintendent. In October 1902 Miss Ryan received from the Matron-in-Chief of Queen Alexandra's Imperial Military Nursing Service notice to send in an application for an appointment to the new service, and she did so in November. In reply to her application she was offered an appointment which she considered was inferior to that which she had in the old service, and she refused it. Ultimately she was retired at the age of 48 on a pension of £34 per annum. These facts were not in dispute, but Miss Ryan, in the exercise of her discretion, sought to recover from the Crown compensation on the ground that she had been prevented from serving under the Crown until she was 60 years of age, when a larger pension would be due to her. Her counsel contended that the nursing sisters and the nursing staff of the Army were not bound either by the decisions which applied to the Military Service, or by the regulations dealing with the Civil Service, and he also submitted that in the alternative his client's services had not been terminated by the pleasure of the Crown, seeing that she had been dismissed from the nursing service contrary to the provisions of the Army Order of July 1902, No. 168, which provided that in the event of a member of the late Army Nursing Service accepted by the Nursing Board as a member of Queen Alexandra's Imperial Military Nursing Service declining to accept the new terms of pay, pensions, etc., she should be allowed to serve upon the terms of her existing engagement. Mr. Justice Bigham, however, expressed his conviction that Miss Ryan's engagement was, like all other appointments, subject to be terminable by the Crown at will, and he added that he was quite satisfied that it had been so terminated. The petition was therefore dismissed, but the Attorney-General forbore from asking for costs against the plaintiff. It is a pity that Miss Ryan, who has had a distinguished career and is the possessor of the Royal Red Cross for services rendered in Egypt and Crete, was advised to bring the action, but the judgment may be useful to army nurses who are not aware that the Crown has full power to terminate appointments at will.

### THE SUPPLY OF ARMY NURSES.

At the meeting of the British Medical Association at Oxford last week Surgeon-General Cuffe, who took part in the discussion in the Navy, Army, and Ambulance Section on the "Organisation of Civil Medical Aid during Peace, so as to ensure an immediate reserve of trained assistance on the outbreak of war," said that "the difficulty was to obtain trained male and female nurses who would be available at any time for service at home or for war abroad." It is not, of course, suggested that either male or female nurses are unwilling to undertake war duty. The truth is that they cannot afford to relinquish permanent for temporary employment. The only remedy appears to be the maintenance of an adequate permanent staff, but this would involve a largely increased expenditure, which we are afraid neither the Government nor the nation is prepared to sanction.

### TRAINING FOR MIDWIVES AT BIRMINGHAM.

For 60 years the Birmingham Lying-in Charity has quietly been doing its work amongst the poor of that city. It has given assistance during that time to 32,000 women. Now, in view of the new order of things brought about by the Midwives Act the committee of ladies attached to the institution are attempting to raise a fund to build and equip a central home, from which the work can be carried on and where midwives can be trained to supply the places of those self-trained, and mostly ignorant, women who, failing to qualify themselves by 1910, will be obliged to retire from the ranks of midwives altogether. The site for the building has been already secured in Loveday Street and has been paid for, but about £5,000 is still wanted to build and equip a suitable home capable of receiving 24 cases where a matron and two resident midwives will be always in residence to help and instruct the probationary nursing staff. In the six districts it is hoped to give annually efficient training to at least 12 women. But unless the necessary funds are provided by voluntary effort the ladies of the charity affirm, and probably with truth, that "the charity may languish and perhaps become extinct." This is not, however, likely to happen in Birmingham.

### THE EMPLOYMENT OF VILLAGE NURSES.

It will be seen from a report in another column that Miss Amy Hughes, superintendent of County Nursing Associations, has given our Commissioner a very clear and interesting account of the work of that organisation. Miss Hughes makes an excellent defence of the employment of village nurses under the auspices of the Association. It is not pretended that these village nurses are fully trained, or that they are able to discharge the duties of fully-trained nurses. The period of training is for six, nine, or twelve months. Midwifery is included, and the village nurses are always under the supervision of the county superintendent, who is, of course, a fully-qualified Queen's nurse and midwife. This, as Miss Hughes says, is the primary point; and, so long as the conditions which she enumerates are observed, we do not think that either fully-trained nurses or the public can in any way suffer from the existence of a carefully organised minor order.

### A JOHANNESBURG APPOINTMENT.

We understand that Miss Waller has been appointed matron to the New Primrose Hospital at Johannesburg. She was trained at the Bradford Royal Infirmary during the period when Mrs. Magill, the present lady superintendent of the Johannesburg Hospital, was matron of that institution. Subsequently, Miss Waller was charge nurse at the Western Fever Hospital, London. In 1896 she went to South Africa and entered the Johannesburg Hospital. During the war in South Africa she served for a year as a member of the Army Nursing Service Reserve. The salary attached to Sister Waller's new post is £180 a year, and the New Primrose Hospital is pleasantly situated six miles east of Johannesburg in the midst of a gold-mining district. Nurses who received their training at the Bradford Royal Infirmary will be glad to hear of the promotion of one of their own training school.

### THE REGISTRATION QUESTION IN AMERICA.

In connection with the registration question, we learn that the managers of the Hahnemann Hospital in New York contemplate deciding not to register their trained nurses under the new State law which permits those who meet its requirements to use the letters "R.N." after their names and which provides for the registration of training schools and nurses. It appears that they fear that the stringent educational standard would debar many desirable candidates from applying for admission to the training school. The nursing staff at the Hahnemann Hospital consists of three graduates and 24 pupil nurses.

### TRAINING AT ADDENBROOKE'S HOSPITAL.

An interesting syllabus of lectures and teaching at Addenbrooke's Hospital, Cambridge, has been prepared, and will in future form the basis of training. First year probationers are to receive eight lectures on elementary anatomy and eight on elementary physiology. They are also to be instructed in the processes of fermentation, and their relation to sepsis, infection, and antiseptics, as well as taught in the wards. The lectures to the second year probationers will deal with elementary surgery in relation to nursing, and with elementary medicine in relation to nursing. There will also be teaching in the wards on a number of highly important nursing duties. Lectures on general nursing are finally to be given by the matron, who also teaches all the nurses bandaging, and the third-year nurses are to be taught nursing and general ward management, how to train and teach probationers, the care of ward linen and stores, the ordering of stores and of the dietary of the patients, with such duties as will enable them to take a ward sister's part if they prove suitable at the end of their training.

### NURSES' QUARTERS AT THE HOLBORN INFIRMARY.

An attempt was made at the last meeting of the Holborn Guardians to rescind a resolution which was passed on November 25th last year, to defer the question of improving the nurses' quarters at the infirmary for two years. Miss Baker, who was one of the supporters of the motion for rescission, said that if the Guardians went into other institutions of the kind, they would find the nurses' quarters far more comfortable than those at the Holborn Infirmary. In reply to the contention that the number of inmates is on the decrease, she maintained that the staff of nurses ought to be kept up, and urged that if the Guardians wished to secure a good class of nurses, they must give them accommodation equal to that provided elsewhere. On a division, however, the rescission was opposed by 17 to six votes, and nothing will therefore be done, at the earliest, until the winter of 1905.

### HOSPITAL NURSES AND STREET DUTY.

In connection with the Royal visit to Liverpool a statement was made in one of the local papers that "three hundred nurses were sent from the Liverpool hospitals to do street duty." If this had been the case the inevitable conclusion would have been that the Liverpool hospitals are greatly over-staffed. Moreover, it is not the business of hospital nurses to undertake street duty. As a matter of fact, the work along the line of route was done by the members of the St. John Ambulance Brigade. It was not of a serious nature, nor were the services of fully-trained nurses required.

### IMPORTANT CHANGES AT WHITEHAVEN INFIRMARY.

It has been decided by the Governors of the Whitehaven and West Cumberland Infirmary, after careful consideration, to make important changes which involve the retirement of Mrs. Hellon, who has been matron for 27 years. As Mrs. Hellon is to receive a pension of £50 a year, it cannot be said that the Governors are treating her badly, and she does not make any complaint. In future the matron will, however, require to be a fully-trained nurse, and though Mrs. Hellon has admittedly been a faithful and efficient servant, she does not fulfil this essential condition. The new matron is to have a staff of not less than two fully-trained nurses, and six probationers, and the person who has acted as male nurse is in future to devote himself entirely to the duties of dispenser, porter, and packer. The new arrangement will come into operation on January 1 next, and we congratulate the Governors of the infirmary upon their determination to move with the times.

### A CHAIRMAN'S THOUGHTFULNESS.

Among the recipients of Pension Fund certificates from the Queen at Buckingham Palace were the matron of the Queen Victoria Nursing Institution at Wolverhampton, the matron of the Wolverhampton and Midland Counties Eye Infirmary, and eight nurses. The contingent from Wolverhampton were thoughtfully provided with dinner in the dining-car of the train on their return journey, by Mr. G. N. Adams, chairman of the Queen Victoria Nursing Institution, to which the eight nurses are attached. His kindness was much appreciated.

### AMERICAN VISITING NURSES IN CONFERENCE.

A number of American nurses attended a special conference of visiting nursing associates at Portland, and some very interesting questions were raised. An animated discussion arose on the issue, "Should the educational and instructive features of visiting nursing be given such importance as to make skilled nursing only of secondary consideration?" and the prevailing view of those who took part in it were that nursing should distinctly not be a secondary consideration, but that proper instruction should be combined with it for the sake of the families, as well as for that of the patients themselves. The danger of over-emphasising instruction to the obliteration of the original conception of visiting nursing—the trained nursing care of the sick poor—was dwelt upon. Another question was, "What are the best methods for a visiting nurse to pursue in order for her to secure sufficient care for her very sick patients at night?" and it transpired that the methods in force in most cities of the United States are identical. Caretakers are employed where possible, and at Baltimore suitable women have been trained by means of special classes and demonstrations for this work. "The advisability of impressing upon the public the growing necessity of nurses devoted to the care of tuberculosis" was debated at length. Miss Damer, of New York, gave an account of work done in connection with Bellevue Hospital and by the Board of Health, which employs eight nurses in this special district work and three in connection with special dispensaries. There was a notable difference of opinion upon the point whether district nurses should live in the district in which they work or where they choose, the preponderating view being that they should live among the conditions which they seek to ameliorate. It was strongly contended that central homes are advantageous in affording opportunities for conference and direction, and providing better quarters for the nurses than an ordinary boarding-house. Several speakers were in favour of a prescribed street uniform, but others maintained that the less conspicuous the dress of a nurse the better.

### CHESTERFIELD GUARDIANS AND DISTRICT NURSES.

It cannot be said that the Chesterfield Guardians received with much sympathy a report from their own committee who recommended that £5 per annum be subscribed for the provision of a district nurse for Chesterfield and a like sum for Staveley. A motion that the report be simply received and the committee thanked for their services, proposed by a guardian who said that he should have liked to add "that further consideration be deferred until this day twelve months," was carried, and nothing therefore will be done at present. We observe that one of the opponents of a contribution expressed the fear that if the guardians gave assistance, a certain number of voluntary subscribers would withdraw their support from the Nursing Association. If we thought this we should not advise the guardians to render assistance; but the invariable experience of the managers of district nursing associations is that grants of the kind deprecated by some of the members of the Chesterfield Board have a stimulating effect and tend to increase the number of voluntary subscribers.

### DISTRICT NURSING AT COLCHESTER.

The annual meeting of the Colchester District Nursing Association was held, not in a stuffy room, but, by invitation of Miss Round in the pleasant grounds of The Holly Trees. The Mayor, who was one of the speakers, said he trusted that before long a home for the nurses would be established, but at present the finances required to be put on a sound basis. There was a balance of £28 15s. 7d. to the good at the beginning of last year, and this has been exhausted. Meanwhile, the work of the nurses was increasingly appreciated. After the report had been adopted, Miss Honnor Morten delivered an address on nursing, in the course of which she referred to the insanitary conditions prevailing amongst the poor, and urged that one cause of the deterioration in the national physique was the cramming of school children with lessons on numerous subjects at an age when they were not strong enough to stand the strain.

### SHORT ITEMS.

The Children's Home at Much Hadham, Herts, has sustained a severe loss by the death, in London, after an operation, of Miss Elise Byrne Hoskier (Sister Elise), who was laid to rest at West Wickham on July 26.

### The Nursing Outlook.

"From magnanimity, all fear above;

From nobler recompense, above applause,

Which owes to man's short outlook all its charm."

### REGISTRATION PROS AND CONS.

Now that the Select Committee has risen for this session, we have several months before us in which to consider the difficulties with regard to the registration of nurses which have already been made apparent.

First of all let us look at Mr. Walshe's view with regard to male nurses. Mr. Walshe was strongly in favour of registration of male nurses, but he had no suggestions as to where the training of the nurses could take place that were practicable. He apparently would be content if those male nurses who hold the certificate of the Medical Psychological Society were allowed to register; but as this certificate is no criterion of general or surgical nursing, it would be impossible to put its holders on the same footing as the holders of the Charing Cross certificate, for instance. Here is one difficulty which must be faced: if male nurses demand to go on the register, it is only fair that thorough general training should be provided for them.

Miss Amy Hughes gave her evidence remarkably well, but her scheme is purely voluntary and for three-year nurses only, and it is difficult to see how it would be any more effective than the present register of the Royal British Nurses' Association Miss Hughes said that at most one third of the nurses now working would join; and what is really needed is not to help the best third, but to raise and control the second third, and to eliminate the third third! Miss Hughes instanced the case of a nurse who having had two years' fever nursing then applied for a responsible post in general nursing, and was astonished that she was not accepted. Miss Hughes seemed to think that this woman had been deluded; but surely the delusion was her own, and the want of comprehension she showed proved her such a dullard as it is undesirable to register? Miss Hughes' strongest point was the reading of three letters from hospitals at which she had made inquiry about nurses who held their certificates; in each case the reply was that no record of the nurse was kept after the certificate was given, and therefore the inquiries could not be answered. The Committee also seemed impressed with Miss Hughes' description of the work of an inspector of nurses; it was obviously a new idea to them that an inspector could go round and see the nurses at work.

Two of the medical men on the Committee showed their own position so far as they had considered the question at present. Dr. Hutchinson is evidently in favour of registering not the nurses, but the nursing institutions which supply the nurses. He would license the different homes and co-operations and make them responsible for the private nurses whom they send out. This has the advantage of being practicable, but it has the disadvantage of putting the individual nurse more than ever under the thumb of the "superintendent" or "secretary," and is certainly not what those nurses desire who have signed in favour of registration. Dr. Hutchinson spoke very kindly of nurses in general and mentioned that in many years' work with them he had come across very few bad ones. He is evidently open to support any scheme for the bettering of the nursing class that is sensible and possible. Dr. Ambrose, on the other hand, stated his belief that registration was only being supported by those societies that hoped to have representation on the central council and thus add to their own importance. To quote his words, "It is not the public, it is these bodies who want to dominate the scheme, who are asking for registration."

So far then as the evidence has gone at present it seems to indicate that registration is only suggested as a sort of added glorification for the nurse who has trained three years in a large and important hospital and enjoyed all possible advantages. Or else it is to be the licensing of nursing homes and the further subjection of the individual to the institution.

Now, the nursing scandals have almost invariably been connected with those nurses who do not work from institutions, and with those who have not had their three years' training. Any scheme for raising the status and training of nurses, must include nurses as a body. The object of legislation should be to stop the six-weeks trained maternity nurse from undertaking an appendicitis case, or to keep the cottage nurse from undertaking important surgical work and burning the patient. This is what the public really needs: it needs a legal minimum of training which should be demanded of every nurse—male nurses, mental nurses, maternity nurses, or cottage nurses; all these are liable to get complicated cases, and all should have had at least one year's experience and teaching in general nursing, be examined in it, and until they have passed in it be ineligible for any higher training. Thereafter the would-be mental nurse can go to an asylum, and the surgical nurse to a hospital, and at the end of another two years take any sort of honours examination, or go on any sort of glorified roll they like. But any Act of Parliament must be for the benefit of nurses as a whole, and the public as a whole: it must not be for the favoured few.

### Lectures upon the Nursing of Infectious Diseases.

By F. J. Woollacott, M.A., M.D., B.S.Oxon, D.P.H., Senior Assistant Medical Officer, Park Hospital, Metropolitan Asylums Board, Hither Green.

### LECTURE II.—THE PREVENTION OF INFECTIOUS DISEASES.

The prevention of infectious diseases is a subject of vast importance, and great and increasing attention is being paid to it all over the world. In this country an immense amount of work has been done with, on the whole, very satisfactory results. Efforts have been made in two directions. First, it has been necessary to keep a close watch on the various agencies by which disease is known to spread, and so to detect and remove any source of mischief. In this way the water-supply has been rendered pure and wholesome, the removal of sewage has been greatly improved, and food has to a large extent been kept free from contamination. In the second place efforts have been made to improve the general health of the population. Overcrowding in dwelling-houses has been checked, and insanitary work-places rendered illegal. In some towns the worst slums have been pulled down and airy and well-built dwellings erected in their place. These measures have undoubtedly been of value, for a healthy, well-fed, and well-housed person is better able to ward off the attacks of disease than the half-starved dweller in the slums. The effects on the spread of infection have been considerable. Typhus and relapsing fever have almost disappeared from this country, while enteric fever has much diminished. In the case of other diseases, such as scarlet fever and diphtheria, the results have been less satisfactory, and at present the most useful preventive measures are the isolation of the sick and the disinfection of their surroundings. These two measures must now occupy our attention.

### I. Isolation.

By this is understood the separation as complete as possible of the sick from the healthy. It may be attempted in private houses, though often ineffectually; but, on the whole, there is a growing tendency to build special hospitals for the purpose. These are already thickly dotted over this country, and their number is steadily increasing. The diseases for which accommodation is usually provided are scarlet fever, diphtheria, typhus, enteric fever, and small-pox; and a large proportion of their victims now avail themselves of this provision. In London, for example, about three quarters of all scarlet fever and diphtheria cases are removed from their homes, and nearly all the small-pox. It is possible that in the future it may be found advisable to treat more of the infectious diseases in hospital. The chief difficulty in the way is the expense entailed. Some of the others, measles for instance, are quite as easily spread and quite as dangerous as those just mentioned; so that in these cases an attempt should always be made to render the home isolation as thorough as possible. It must be admitted, however, that some of these diseases do not show a great tendency to spread. Enteric fever is an example. It is still received into many general hospitals. The patients are treated in the ordinary wards, and rarely, if ever, do other patients suffer. The reason probably is that the chief source of danger is in the stools and the urine, which, with proper precautions, can be disposed of safely. That the disease is very definitely infectious, however, is proved by the fact that members of the nursing and medical staff are occasionally attacked. The advantages of special hospitals for the treatment of the infectious sick are numerous. In the first place complete isolation is ensured throughout the illness. Visiting is usually not permitted, so that the patients have no opportunities of seeing and possibly infecting their friends. The hardship of this restriction is counterbalanced by the better conditions under which they are placed in airy wards and with skilled attendance. The convalescent period, too, is rendered more pleasant and healthy, as the exercise grounds attached to the hospitals enable the patients to pass much of their time in the open air. The attempt to isolate in ordinary dwelling-houses is rarely satisfactory from the patient's point of view. He is confined to one room, usually in an out-of-the-way part of the house, and exercise in the open air is difficult to obtain.

We sometimes hear it said that children are sure to catch most of the common infectious diseases sooner or later, and therefore it is hardly worth while to take elaborate precautions. This sounds plausible, and it must be admitted that precautions often fail. On the other hand they often succeed, and in any case we should bear in mind that as children grow up, every year renders them less susceptible to infection and more likely to recover if attacked. The first few years of life are the time of greatest danger, and every effort should be made to tide them over in safety. Scarlet fever, for example, is more than three times as fatal in children under five years of age as in those between the ages of five and ten, while later in life the risk is very slight.

### II. Disinfection.

Under this head are grouped all the agencies by which infection is destroyed. It is obvious that unless this be done the full value of isolation is not obtained, for though the patient is prevented from doing harm directly, the articles leaving his bedside might still spread the disease. We saw in the last lecture that infection implied the presence of living organisms. The object of disinfection is to kill these organisms. Anything short of this is unsatisfactory. They never give quarter themselves, so we must modify the golden rule, and treat them as they would treat us. Before considering the artificial methods of disinfection, mention may be made of what may be called the natural method. It is a curious fact that bacteria do not thrive in the presence of direct sunlight, and sooner or later die off. The importance of this is great, for the sun's action is far more widespread than any of man's appliances; and advantage may be taken of it when other means fail, by exposing infected articles in the open air to the sun's rays. Unfortunately these rays do not penetrate very deeply below the surface so that their effect on bulky articles is very slight, while in this climate they are often not available when wanted. We must rely, therefore, on other methods, of which two are in common use—(*a*) the application of heat; (*b*) the use of chemicals.

(*a*). The simplest and most efficient way of destroying infection is to burn the infected articles. Obviously this remedy would be too expensive for general application, but within limits it is useful. Old books, toys, or worn out clothes for example are best disposed of in this fashion, and it may be here mentioned that it is a good rule not to take into the sick-room any article of value which is likely to be injured by the disinfection that will be necessary subsequently. Fortunately the services of heat can be utilised in a form much less likely to cause damage, viz., in the moist state, as boiling water or steam. Used in this form, heat is by far the best disinfecting agent at our disposal. Its action is very thorough and it is capable of very extended application. It is almost invariably used at the present time in sterilising surgical instruments and dressings. In cooking, too, its value is seen, and a sure way of rendering harmless milk or water containing the bacteria of disease is to boil them for a few minutes. The disinfection of clothing should as far as possible be effected in the same way. Linen and cotton garments may be boiled as in the ordinary process of washing. Woollen articles, however, such as blankets and flannels would be destroyed by this process and must be submitted to the action of steam in what is known as a steam disinfector. This consists of an air tight chamber with a door at each end. The infected articles are brought in through one door and the chamber is closed up. Steam is then admitted, usually under pressure, and allowed to act for about half an hour. The contents of the chamber are then dried and removed through the other door into a room which is kept free from infection. In this fashion bulky things, such as mattresses and pillows, are also disinfected, the great value of the method being due to the fact that the steam has great penetrative powers, so that, however deeply the microbes may have hidden themselves, it follows and destroys them. Such an apparatus as that just described is in the possession of every well-managed district in the country, and is used to supplement the simpler domestic methods. Hospitals, of course, have steam disinfectors of their own. Clothing or bedding soiled with excreta should be steeped for some time in water to remove the grosser impurities and then boiled or steamed. In enteric fever this must be done very thoroughly.

In some few cases neither steam nor boiling water can be made use of. Leather articles, for example, would be irretrievably ruined by either, so it is best to expose such things to the action of hot air in an apparatus somewhat similar to the steam disinfector. The degree of heat must, of course, be regulated, otherwise there would be risk of scorching or even charring. For this reason the domestic oven is unsuitable. Hot air is far less efficient than steam, and should be employed as little as possible.

(*b*) Chemical disinfectants are now much less relied on than formerly as they are somewhat uncertain in their action. To be effective they must be used in strong solutions and allowed to act for a considerable time. Most, if not all, of them are very poisonous, so that they must be carefully looked after. For nearly every purpose they are inferior to heat in some form or another. The disinfectants most commonly employed are carbolic acid and perchloride of mercury or corrosive sublimate. Many others might be mentioned, but for the most part their germicidal action is very slight. They probably have considerable influence in hindering the growth of bacteria, but are not powerful enough to destroy them. In other words they are antiseptics and not true disinfectants. Many of them serve the useful purpose of removing offensive smells, and are therefore called deodorants. Condy's Fluid is a well-known example of this class. Disinfectants are occasionally used in the gaseous form. This is known as fumigation. The chemicals usually employed for the purpose are burning sulphur, and less frequently chlorine gas and formalin vapour.

### How to Become a Midwife: The Rules of the new Act Explained.

By E. L. C. Appel, M.B., B.S., B.Sc.(Lond.).

(*Concluded from page 232.*)

*Notification of Cases Outside her District.*

If a midwife who resides and carries on her practice in a district should at any time practise or act as a midwife in any other district than her own, then she must within 48 hours at the latest after she begins so to practise or act send a like notice (on Form 12) to the Local Supervising Authority of that other district, and give the date and address at which she commenced to practise or pursue her calling in that other district.

The following shows how, in these cases, Form 12 should be filled in and signed:—

Midwives Act, 1902, Section 10.

To the Local Supervising Authority of * the Administrative County of Cheshire.

I, Mary Anna Smith

*(*formerly*) Mary Anna Thomas ..............................(C.), holding a certificate from the Central Midwives Board, No. 15, dated the 5th of August, 1903, hereby give you notice that on the 10th day of February in this year I acted as a midwife at Riglington, within your area.

(Signed) Mary Anna Smith.

Residing at 18 High Road, Duddleswell, and pursuing my calling at 18 High Road, Duddleswell.

Dated this 10th day of February, 1904.

* Strike out words not applicable.

*Notification every Year in January.*

If a midwife has been in practice in a district, and intends to continue to practise in that district, she must in the month of January in every year during which she continues to practise in that district send to the clerk of the Local Supervising Authority in her district a notice on Form 12, similar to the one she sent in when first she began to practise in that district.

*Notification of Deaths.*

If, in her practice as midwife, a mother whom she is attending or the child should die before a doctor has been in attendance at that case, the midwife must, as soon as possible after the death, notify the death to the Local Supervising Authority of the District.

See Clause 18 of the Regulations.

*Notification of Stillbirth.*

In all cases of stillbirth occurring in a midwife's practice, when a doctor is not in attendance, the midwife must, as soon as possible after the occurrence of the stillbirth, notify the stillbirth to the Local Supervising Authority of the District.

See Clause 18 of the Regulations.

*Notification of Puerperal Fever and other Infectious Diseases.*

If a case of puerperal fever or other infectious disease should occur in a midwife's practice, she should at once send for medical help (using the proper form), and she must within 12 hours send a copy of this, by post, to the Local Supervising Authority of her District. She must also keep a copy for herself.

See Clauses 18 and 19 (*b*) of the Regulations.

The following is an example of the way in which the form for medical help should be filled in:—

No. 3. Date, March 4th, 1904.
Name of Patient, Mrs. Jackson.
Address, Ivy Cottage, Hickworth.

Requires medical assistance at once on account of fever.

(Signed) Jane Brown (Certified Midwife).

Sent to Dr. Robinson, at The Grange, Hickworth.
Time of sending message, 2 p.m.

After seeing a patient who is ill with puerperal fever or

### HOW TO BECOME A MIDWIFE—*Continued.*

some other infectious disease, the midwife must not visit any of her other patients nor attend another labour case till she has complied with all the requirements of Clause 5 of the Regulations.

*Notification of Medical Help Sent For.*

When a midwife requires medical help for a case which she is attending she must send a written message to the doctor, giving the name and address of the patient, the reason why medical help is required, and the time at which the message was sent. She must send a copy of the message by post to the Local Supervising Authority, within 12 hours, and must keep a copy for herself.

The following is an example of a message asking for medical help for a case where the lying-in woman was seized with a fit during the first stage of labour:—

No. 6. Date, March 18, 1904.
Name of Patient, Mrs. Freeman.
Address, 1 Willow Street, Hickworth.

Requires medical assistance at once on account of a fit.

(Signed) Jane Brown (Certified Midwife).

Sent to Dr. Robinson, at the Grange, Hickworth.
Time of sending message, 6 A.M.

*Notification of Change of Address.*

A midwife who removes to another house or place of residence should at once send notice of her change of address to the local supervising authority.

*Inspection of Midwife's Case-book, Appliances, etc.*

It is the duty of the local supervising authority to make arrangements for the inspection of every midwife's case-book, bag of appliances, etc.

The purpose of this inspection is to make sure that the rules and regulations are being properly carried out.

As regards her case-book, the midwife should keep this written up daily, so that it is always ready for inspection.

The bag in which she keeps her appliances, etc., should be used for no other purpose. Instruments or appliances should be washed directly after use, before they are put back into the bag.

Her place of residence and mode of practice may also be inspected and investigated when the local supervising authority think it necessary.

*Suspending a Midwife from Practice.*

It is the duty of the local supervising authority to suspend a midwife from practice if she fails to carry out the directions and rules laid down to prevent the spread of infection.

A midwife must, therefore, be careful

I. To use disinfectants as directed by the regulations, and to be very clean in her person and in her work, in order to avoid the possibility of causing illness or fever in any of the lying-in women she attends.

II. To *at once* discontinue visiting and attending any other patients if she suspects or knows one of her patients to be suffering from puerperal fever or other infectious disease.

III. To *at once* report a case of puerperal fever or other infectious disease to a doctor and to the local supervising authority. If the midwife thinks a woman is probably suffering from puerperal fever or other infectious disease, but is not quite sure, it is best to call in a doctor at once, and let him decide whether it is a case of fever or not.

The local supervising authority have power to suspend the midwife from practice, and will at once report the suspension, and the reasons for the suspension, to the Central Midwives Board.

*Malpractice, etc.*

Charges of malpractice, negligence, or misconduct on the part of any midwife will be investigated by the local supervising authority of the district in which the midwife carries on her practice, and if proved against her will be reported to the Central Midwives Board.

*Conviction and Prosecuting of an Offence.*

If a midwife is convicted of an offence, the local supervising authority of the district in which the midwife carries on her practice will report the conviction to the Central Midwives Board.

Any offences under the Midwives Act punishable on summary conviction may be prosecuted by the Local Supervising Authority.

### APPENDIX.

Approved Institutions under Section C of the Rules of the Central Midwives Board (see p. 180).

(*Preliminary List.*)

Queen Charlotte's Lying-in Hospital, London.
Liverpool Ladies' Charity and Lying-in Hospital.
Manchester Southern and Maternity Hospital.
British Lying-in Hospital, London.
Newcastle-on-Tyne Lying-in Hospital.
General Lying-in Hospital, York Road, Lambeth, London.
Glasgow Maternity Hospital.
Dundee Maternity Hospital.
District Nursing Association, St. James's Square, Cheltenham.
Maternity Charity and District Nurses' Home, Plaistow, London.
National Maternity Hospital, Dublin.
Edinburgh Royal Maternity Hospital.
City of London Lying-in Hospital.
Birmingham Workhouse Infirmary.
Bristol Royal Infirmary.
Brownlow Hill Workhouse Hospital, Liverpool.
Essex County Cottage Nursing Society.
Gloucester District Nursing Society.
Hull Lying-in Charity.
Ipswich Nurses' Home.

Institutions whose Certificate is Accepted as an Approved Qualification under Section 2 of the Act (see p. 164).

(*Preliminary List.*)

Queen Charlotte's Lying-in Hospital, London.
Liverpool Ladies' Charity and Lying-in Hospital.
Manchester Southern and Maternity Home.
British Lying-in Hospital, London.
Glasgow Maternity Hospital.
St. Mary's Hospital, Manchester.
City of London Lying-in Hospital.
Royal Maternity and Simpson Memorial Hospital, Edinburgh.
Salvation Army Maternity Home and Hospital.
National Maternity Hospital, Dublin.

[These articles are published in book form by the Scientific Press, Ltd., 28 and 29 Southampton Street, Strand, W.C., at 1s., and 1s. 1d. post free. All midwife forms, price 1d. each, postage ½d. extra, can also be obtained from the Scientific Press.]

### The Work of County Nursing Associations.

BY OUR COMMISSIONER.

There is still so much ignorance respecting the work of County Nursing Associations in affiliation with Queen Victoria's Jubilee Institute for Nurses, that it seems important to describe as clearly, and yet as briefly as possible, the conditions under which it is carried on. The most authoritative source of information is usually the fountain head. I therefore called upon the Superintendent of County Nursing Associations at 120 Victoria Street for the purpose of asking Miss Amy Hughes to supply me with all the details of the work.

To start with, it is essential that the precise manner in which a County Nursing Association can be constituted should be understood. It consists of a central organisation of the principal people in any county, who band themselves together with the object of providing either fully-trained nurses or village nurses according to the needs of the local associations.

### The Fully-trained Nurse.

As to the fully-trained nurses, the superintendent stated that they must have three years' training in a general hospital, three or six months' district training, and possess the midwifery certificate of the Central Midwives Board. The County Associations themselves may give both district and midwifery training to hospital-trained nurses, requiring them, in return, to work for a stipulated time wherever they may be sent in the county. In some cases the Queen's Institute will give the training, the nurses being under an obligation to serve the County as Queen's nurses for the same period as the Institute. In fact, they become Queen's nurses for the particular county, and are doubly bound.

"At the end of the one or two years for which they are bound, if satisfactory," continued the superintendent, "they are free to continue as Queen's nurses anywhere, and to serve in the same or some other county. They cease to be Queen's nurses when they cease to work for an association affiliated to the Institute. The cost of these nurses for maintenance after training is from £85 to £100. They receive a minimum salary of £30, and everything in the shape of board, lodging, and uniform is found them. The Associations have been most fortunate in respect to the number of applications they have received from well-trained nurses. If one of the county superintendents advertises there are frequently from eight to a dozen suitable applications. Many of them, it is found, signify a desire to work in a particular county, because they have friends there. The work of these nurses is, in short, similar in all respects to the ordinary Queen's nurses."

### The Village Nurse.

"Then there is the other class—the village nurses—so-called in order to distinguish them from cottage nurses. They were originated for the purpose of meeting the difficulty of rural districts, often thinly populated, and with no means of procuring the necessary funds to defray the cost of a fully-trained nurse. In addition to this difficulty, the fully-trained nurses do not find enough work to induce them to settle down in these quiet country districts, where the birth-rate is often not more than 10 or 12 in the year, and difficult cases are few and far between. Village nurses are chosen from suitable candidates in the county, and are trained both as midwives and in the elements of general nursing. They gain their knowledge in the houses of the people under fully-trained nurses."

### The Age Question.

"The older and steadier women are preferred, and as a rule no one under 24 is engaged. Midwifery and maternity nursing is their special work. The village nurses are drawn from among the daughters of small farmers, veterinary surgeons, and small tradespeople; occasionally superior domestic servants, who seem naturally qualified for the work, are selected. The training of many of these nurses is paid for by grants from the Technical Education Committee of the County Council, or by funds collected in the county on behalf of the County Nursing Association."

### The Scholarships.

"The three County Councils in Lincolnshire (Holland, Kesteven, and Lindsey), the County Councils of the Soke of Peterborough, Nottinghamshire, Hampshire, Cornwall, Sussex, and Cumberland give directly to the county associations; and, in addition to these, which are affiliated to the Queen's Institute, scholarships for the same purpose are offered by the Northumberland, Norfolk, Suffolk, Bedfordshire, Essex, Wiltshire, and Shropshire County Councils; also by Flintshire. As to the cost of training the village nurse, it is £35 for six months, and from £45 to £50 for nine or twelve months. This includes travelling expenses, outfit, uniform, and sometimes pocket money. It will be necessary very shortly to extend the period from six to nine months, owing to the longer time required under the provisions of the Midwives Act for midwifery. The training for midwifery and also for district nursing is given at the Plaistow Home, which in April last was affiliated to the Queen's Institute."

### The Standard of Payment.

"The village nurses, in return for their training, are bound to the county associations for three years, at an inclusive salary corresponding in amount to the average weekly wage of the labourer in the county. About 14s. or 15s. may be taken as the standard. With few exceptions these nurses are supported on the benefit system, and this standard was decided upon because the subscribers are chiefly of the labourer class who would not understand the nurse being paid more than that on which they keep themselves, their wife and children. The average annual cost of the village nurse to the local association is from £45 to £55. This covers the cost of a bicycle and the renewal of uniforms."

### The Importance of Supervision.

It will thus be seen that the County Nursing Association provides two distinct classes of nurses trained according to two grades, and fully equipped, to the local association, which, in return, is required to maintain them and provide uniform, nursing appliances, and whatever may be essential. But there are other features of great importance, and as the superintendent said:—"The primary point with regard to the village nurses is that they are under constant supervision by the county superintendent who is a fully-qualified Queen's nurse and midwife. The county superintendent also visits the Queen's nurses working in local associations affiliated to the County Association from time to time. She has not only to keep the village nurses up to the mark, but to teach them more. When a village nurse is employed, the county superintendent will herself also attend. In the event of an operation being necessary, all nurses, whether fully trained or village, have to report to the county superintendent once a month, and notify the new cases. In serious cases she renders them help.

### Why Village Nurses are Needed.

"One of the conditions of the agreement which is made between the County Association and the Queen's Institute is that the employment of village nurses for general sick nursing is undertaken entirely upon the responsibility of the association under which they are engaged, and with the direct sanction of the medical men. Village nurses were, in the first instance, intended only for midwives and maternity nurses; but it was found impossible to prevent them from nursing the sick when desired to do so by the doctor. In fact, sufficient financial support could not be obtained if they were not allowed to do some general nursing."

County Superintendents.

The duties of the county superintendent being another point respecting which there is a good deal of ignorance, Miss Hughes explained that she not only has to superintend the nurses, but to interview and engage them, subject to the approval of the committee, to arrange their future nursing and sphere of work, to organise the new local organisations, explaining the lines of work, and helping to initiate them, and send reports concerning them half-yearly to the Institute. The county superintendent is herself inspected from headquarters, and an inspector visits every Queen's nurse connected with a County Association once a year. The mention of Lincolnshire as the first county to start a County Association in 1893 led me to suggest that it would be useful to show how an Association is organised.

How to Organise.

"The preliminary step is to hold drawing-room meetings, and then," continued the superintendent, "a public meeting to formally determine whether an Association shall be formed. The next move is to choose as good and representative a general committee as possible, including the medical men of the county. The committee should be able to delegate their powers to an executive committee, which should include a proportion of representatives from the affiliated local associations. There should be a president, vice-presidents, an honorary secretary, an honorary treasurer, and a chairman of the executive committee. When the Association has been formed it applies for affiliation to the Queen's Institute, and the usual agreement is signed. As a rule the local Associations pay an affiliation fee to the Central County Committee."

Ways and Means.

"With regard to ways and means, the financial basis of a County Association should be annual subscriptions. In order to provide grants for training village nurses; for midwifery and district training to certificated nurses; towards the maintenance of a county superintendent; for travelling expenses, postage, printing, and a nurse for emergency or holiday duty, an annual income of between £300 and £400 is indispensable, whether the County Council grants are included or not. The Queen's Institute makes a grant of £50 a year towards the salary of a county superintendent, on condition that the work is in accordance with the standard of the Institute. The General Committee of a County Association ought to help poor districts by making grants to start nurses, and it is advisable that a certain sum should be given out of the central fund to enable nurses to secure, or retain, a pension, as in Nottinghamshire, either in connection with the Royal National Pension Fund or with the National Friendly Deposit Society."

The Benefit System.

In conclusion, the superintendent insisted that if the village nurses do not profess to be "trained nurses," yet they fill a gap.

"They are preferable to the half-trained, undisciplined women who style themselves 'hospital nurses,' and can train in hospital at the end of their agreement. They are only employed in a district at the suggestion of the county superintendent when she finds that it is impossible, on account of the small population, to supply a Queen's nurse, or under a Queen's nurse, generally for maternity work. They are not forbidden to live in the homes of patients, but are allowed to do so under special circumstances. Although payment by fees is disapproved, except for maternity work, there is no objection to the Provident System. Under this system a regular annual subscription is paid as to a club, which entitles subscribers to the nurse's services. A special charge is made for midwifery cases, but maternity nursing under a doctor is frequently given without extra charge. In many manufacturing districts a regular sum is deducted from the weekly wages for the support of the Nursing Association, and supplemented by a house-to-house collection.

Such methods are proved to be more successful than the payment of fees for visits, which tend to limit the usefulness of the nurse by enabling the patient to dispense with her services, on the score of expense, before such a dismissal is desirable."

### Everybody's Opinion.

[Correspondence on all subjects is invited, but we cannot in any way be responsible for the opinions expressed by our correspondents. No communication can be entertained if the name and address of the correspondent are not given as a guarantee of good faith, but not necessarily for publication. All correspondents should write on one side of the paper only.]

### THE RECEPTION AT BUCKINGHAM PALACE.

"A Nurse" writes: As one of the nurses at her Majesty's reception on July 22nd, I was much struck by the amount of extra work such perfect organisation must have meant to the workers at the offices of the Royal National Pension Fund for Nurses, and suggested to Mr. Dick that I wished a 1s. fund could be started among nurses to present those workers with a souvenir of the day. Mr. Dick would not hear of it, but would much like all nurses present to subscribe 1s. extra to the Benevolent Fund this year. I am sure that there were many nurses present who would be glad to co-operate with Mr. Dick in his kind suggestion for helping those sisters of our profession whom he knows are so much in need of help. It will practically show that we appreciated the trouble and kind thought of so many to give us a day to be remembered all our lives.

### THE REGISTRATION OF NURSES.

Miss Isla Stewart, matron and superintendent of nurses, St. Bartholomew's Hospital, writes: I see that in your report of my evidence before the Select Committee of the House of Commons on State Registration I "was understood to say that nurses under the Poor Law would be excluded from the working of the scheme." I made no such foolish statement, and on going carefully through the shorthand notes of my evidence I cannot find any answer which seems capable of bearing this interpretation. I shall be obliged to you if you will insert this letter in the next issue of your journal, that it may be distinctly understood that I have no wish to see the great infirmaries excluded from a privilege they so well deserve.

[The explanation of our representative is that Miss Stewart—whose letter reached us the day after we went to press—did not make herself clearly heard, the chairman having to ask her once to speak more distinctly. On this account the words were qualified by the addition "was understood to say."—Ed. The Hospital.]

"Thyrodo" writes:—Will you spare me a small space in your column, and allow me as a nurse to pass a few remarks on "State Registration," which so many speak of as a "protection to nurses and a safeguard to the public." In my opinion it is just the opposite. I am myself a fully-trained nurse, holding a three-years' certificate from a London general hospital; therefore the Bill, if passed, will neither lessen my work nor raise my salary; but I fear that the nursing standard will be lowered by such a movement. Much more will be thought of the technical examination than the nurse's own personal qualities. All will be made to stand alike because they are the possessors of a three-years' certificate. As regards the general public, since my training I have done a good deal of private work, and I cannot fail to see every day how much more essential to a true nurse are good-temper, sympathy, and patience, than the technical part of her training. Though of course a certain amount of study and examination are needed, it is the doctor's business to diagnose the cases and not the nurse's; it would be considered presumption on her part to suggest doing so, and whenever one hears complaints of nurses, I am sure you will agree with me it is more often due to the nurse's conduct than to lack of skill. I cannot see how registration can prove either a protection to nurses, or a safeguard to the general public. A certain class of people are already very bitter against trained nurses, and speak of them as being unfeeling and only machines. State Registration seems a sure way of turning this taunt into a reality, so that the machines and those who are conscientious and true nurses, in the highest sense of the word, will stand alike with those on the register. Who is to judge the difference? The public will then have cause to cry out, and trained nurses, instead of finding themselves protected, will find the profession lowered and nurses themselves thought less of.

Charles H. W. Bridgeland writes: It was with great interest that I read your report of the business transacted by the Select Committee on the Registration of Nurses, but at the same time I am bound to admit that I feel greatly disappointed to observe that the question of male nurses has not once been considered. The question naturally arises: Are they to be permitted to share in the benefits, which will, I presume, accrue, if the Bill becomes law, or is it a preconcerted scheme on the part of a few of the matrons of our larger hospitals to get the whole of the nursing profession under their sway, and to take away the livelihood of those nurses who have independence enough to hold different opinions to themselves? I note that Sir John Batty Tuke, the newly elected president of the Asylum Workers' Association, is on the Select Committee, and as you state in your leading article he has had "great experience in male nurses," he has a splendid opportunity of doing good service to a deserving body of workers and at the same time earning their heartfelt gratitude. It does not follow that because a "mere man" cannot put on cap and strings and a print dress, he is less capable than his female confière, and one does not often hear of a male nurse doing anything that would tend to bring discredit on our profession; on the other hand, even to put it mildly, "unconventional conduct" on the part of certain female nurses, have appeared only too frequently in recent issues of the Press. I mention this, not from ill-feeling, but because it is quite probable that the question of character would be urged against our claims, and it is just as well to show beforehand that that argument would not hold water. Reducing the question to a personal matter:—I should find it very hard if I could not obtain an engagement because I was not "registered," and yet I have held the post of charge-nurse in two large infirmary wards, and I hold certificates for both general and mental nursing, yet, because I do not happen to be one of the "weaker sex," I am to be adjudged incapable of nursing a case of illness when my testimonials from two medical superintendents expressly state that I am "a thorough and most capable nurse," and, furthermore, I am still considered worthy my place on the staff of one of the best nursing associations in the country. As the scheme for the registration of nurses stands at present it is manifestly unjust and tyrannical. This Bill, if it is drafted out in a proper manner, and all classes of trained nurses of good character are allowed to partake of its benefits, will be one of the best things ever done for the nursing profession, but in its present form is more likely to do harm than good, and instead of uniting the profession, will only serve to increase the dissension, which is even now only too prevalent in our ranks.

[It will be seen in reference to our report last week that evidence on behalf of male nurses has since been given before the committee.—Ed. Hospital.]

### Novelties for Nurses.

**By Our Shopping Correspondent.**

### USEFUL FOR THE HOLIDAYS.

Messrs. Fry the renowned chocolate and cocoa manufacturers have sent some specimens of their staple commodities. It was unnecessary to remind one of their excellence, as the quality of the Fry's chocolates and cocoas is known to all. But I am glad to take this opportunity to suggest to nurses the wisdom of providing themselves with this valuable form of food when they start upon their travels. It is impossible to ensure perfect commissariat arrangements if we leave the beaten track, but a kit containing light and portable fare such as chocolate and cocoa renders the traveller independent of the accidents of travel—a tin of Fry's cocoa and milk will secure a nutritious meal if nothing but hot water is forthcoming from other sources. A cup of malted cocoa or chocolate (supplied in tins) is a splendid forerunner to the day's expedition—and the pocket of the pedestrian should never be without a supply of milk chocolate tablets. If nurse travellers follow our advice they will, I feel sure, return to sing the praises of so useful and pleasant an addition to their travelling equipment.

### AN EXQUISITE PERFUME.

The holiday season is the time when most of us invest in some perfume to refresh ourselves on our travels. Somewhat lacking in enterprise, some of us never venture beyond safe and pleasant eau de Cologne. And no doubt this is wise if we are not acquainted with other perfumes of good quality. To those who are ready to venture into the realms of novelty, I am glad to introduce the most delightful scent I have ever come across. This is "Island Bouquet," supplied by Edgar Dupuy, Commercial Arcade, Guernsey. The mingled perfume of fresh flowers is suggested in a manner I do not think is equalled in any other scent. Although Mr. Dupuy's wonderful perfume is little known to the general public, it is the favourite with some of the highest personages in the land, and it may be that those who cultivate exclusive tastes will not welcome the fact that I am letting the numerous readers of The Hospital into the secret! However, I feel Mr. Dupuy's perfumes deserve wider recognition, and so I do not hesitate to suggest that nurses should send him a post-card asking for his price-list (a very moderate one). He manufactures beside an excellent lavender-water, an extract of verbena and eau de Cologne.

### Presentations.

The Johnstone Combination Hospital, Muirhead, Johnstone.—Miss Lillian Muir, on resigning her post as matron, owing to her marriage, was presented by the nursing staff with silver-topped bottles and a silver card-case, by the maids with a silver box, and by the doctor with a pearl and turquoise brooch.

### "The Hospital" Convalescent Fund.

The Hon. Secretary begs to acknowledge with thanks the receipt of 10s. from Nurse M. R., per *The Southern Daily Mail*, and 2s. 6d. per the Travel Correspondent of The Hospital.

### Appointments.

[No charge is made for announcements under this head, and we are always glad to receive, and publish, appointments. The information, to insure accuracy, should be sent from the nurses themselves, and we cannot undertake to correct official announcements which may happen to be inaccurate. It is essential that in all cases the school of training should be given.]

City of London Union Infirmary, Bow.—Miss M. O. Cumming has been appointed sister. She was trained at the Liverpool Hahnemann Hospital, then became staff nurse at the County Hospital, Newport, Monmouthshire, and has since been theatre nurse at the Children's Infirmary, Liverpool, and charge nurse at the City Hospital, Grafton Street, Liverpool.

Fever Hospital, Melbourne.—Miss Evelyn Conyers has been appointed matron. She was trained at Melbourne Hospital for Sick Children and at the Melbourne Hospital. She has since had partial charge of a private hospital at Albury, New South Wales, and of another at Spring Street, Melbourne. She has also done private nursing in Melbourne and New Zealand, and for the past year has held the post of matron of a private hospital in Melbourne.

Fulham Infirmary, St. Dunstan's Road, Hammersmith.—Miss Agnes Margaret McBean has been appointed sister. She was trained at Southwark Infirmary, East Dulwich, and has since been staff nurse at the National Hospital, Queen Square, Bloomsbury. She holds a certificate for massage and medical electricity.

Highwood Ophthalmic School, Brentwood.—Miss Emily Long has been appointed assistant matron. She was trained at Wandsworth and Clapham Union Infirmary, has done private nursing at Eastbourne, and for the past 16 months has been charge nurse at the White Oak Ophthalmic School, Swanley, Kent.

Littlehampton Cottage Hospital.—Miss E. B. Farrow has been appointed nurse-matron. She was trained at Southwark Infirmary, East Dulwich, and has since been charge-nurse at the Miller Hospital, Greenwich, and night sister at the Croydon General Hospital.

Middle Ward Hospital, Motherwell.—Miss Helena M. S. Thornton has been appointed sister. She was trained at the Brownlow Hill Infirmary, Liverpool, and the City Hospital North, Liverpool. She has since been engaged in private nursing, and has taken temporary duty in the Town's Hospital, Glasgow, and the Royal Infirmary, Edinburgh.

Royal Infirmary, Sheffield.—Miss Mary Jane Wilson has been appointed masseuse. She was trained at Southwark Infirmary, East Dulwich, and has since been staff nurse at the National Hospital, Queen's Square, Bloomsbury. She holds a certificate for massage and medical electricity.

Stroud General Hospital.—Miss E. G. Holmes has been appointed nurse. She was trained at the Queen's Hospital, Birmingham, has been sister at the Ear and Throat Hospital, Birmingham, and sister and assistant matron at the Swansea Hospital.

Swindon and Highworth Union.—Miss Ellen Elizabeth Dowley has been appointed superintendent nurse. She was trained at Wandsworth and Clapham Union Infirmary, where she has since been charge nurse. She has also been charge nurse at Cardiff Union Infirmary, and assistant superintendent nurse at Braintree Union Infirmary.

Watford District Hospital.—Miss Florence Foster has been appointed staff nurse. She was trained at Walthamstow Hospital.

West End Hospital for Diseases of the Nervous System, London.—Miss Catherine E. A. Thorpe has been appointed matron. She was trained at the London Hospital. She has since been sister, and has done assistant matron's duties at the Metropolitan Hospital, Kingsland Road, London.

### TRAVEL NOTES AND QUERIES.

By our Travel Correspondent.

Montreux or Clarens (Stella). These two are practically the same; several villages collectively constitute Montreux. It is almost useless for you to say you want a cheap pension, because the word cheap bears different interpretations according to the depth of our purses. Tell me just what you can afford to pay and I will do my best for you. On the whole I think accommodation in Vevey is on a lower scale.

Rooms in Normandy or Brittany (W. B.). I do not mind the trouble at all, but I fear your young friends want an impossibility if they mean £5 each to cover their journey also, frankly it cannot be done. Brittany is much cheaper than Normandy, and quieter, so choose it of the two. There is not the slightest reason why three well-conducted quiet girls should not stay at an hotel. No one will speak to them unless they wish it. I am always trying to impress upon my correspondents that rooms with attendance hardly exist on the Continent. You must either go to an hotel or pension, take rooms and do for yourself, or have a *bonne à tout faire*, neither of these two last plans being suitable for girls. I think the Bay of St. Malo would be the place for them, but in the height of the season I do not think they would get anything under £5 10s. for the three per week. They could stay at St. Servan, a fine centre for excursions, or at St. Enogat, a hamlet of Dinard. Or, if they prefer something more primitive, Lannion is very charming. Lannion is not on the sea itself, but from it on cycles they can visit all the splendid coast scenery of the north-west promontory. Let me hear further, and whether you like any of these plans.

St. Enogat for a Fortnight (Jean). Not on the sum you name; 6 francs per day is 42 per week, double it and you have 84 which, turned into English money is, roughly speaking, £3 7s. 6d., and there is the journey to be reckoned and hotel tips, to say nothing of excursions and incidental expenses, such as washing and other trifles which always make their unpleasant presence felt. Messrs. Cook issue some cheap tickets to St. Malo, only 24s. return. Write to them and see if dates fit. Ordinarily, second class return fare is £1 19s. 3d. I could arrange something cheaper for you in Belgium if you liked because the journey can be made for a very small sum by taking the Tower Bridge route.

A Week in Brittany (Caen).—Write to Hotel des Bains, Trestraon, Pres de Morlaix. It is a primitive place, and they may not answer, and you would have to risk a visit. Trestraon is only about ten miles from Morlaix, one of the most interesting towns of Brittany. The second class return ticket to St. Malo is rather heavy—see answer to (Jean). If you think Morlaix too far, write to the Maison Ste. Anne, St. Servan, and ask for terms. It is a convent, but there are seldom wearisome rules; the nuns are accustomed to boarders. Pension Née, 54 Rue de la Constitution, Avranches, would be a suitable place, I think. Avranches is at the junction of Brittany and Normandy, and looks over to fascinating Mont St. Michel, which is from there an easy excursion.

Rules in Regard to Correspondence for this Section.—All questioners must use a pseudonym for publication, but the communication must also bear the writer's own name and address as well, which will be regarded as confidential. All such communications to be addressed "Travel Editor, 'Nursing Section of *The Hospital*,' 28 Southampton Street, Strand." No charge will be made for inserting and answering questions in the inquiry column, and all will be answered in rotation as space permits. If an answer by letter is required, a stamped and addressed envelope must be enclosed, together with 2s. 6d., which fee will be devoted to the objects of "The Hospital" Convalescent Fund. Any inquiries reaching the office after Monday cannot be answered in "The Hospital" of the current week.

### Echoes from the Outside World.

### Movements of Royalty.

The King and Queen visited Chichester on Saturday after they left Goodwood, and received a loyal address from the Mayor and Corporation. Their Majesties, with whom were the Prince of Wales and Princess Victoria, received an enthusiastic greeting as they proceeded through the gaily decorated streets of the city. Proceeding to Portsmouth, their Majesties and the Royal party subsequently went on board the yacht, which was in readiness in the harbour, and steamed out about two o'clock, arriving at Cowes about six. The King, Queen, and other members of the Royal family attended Divine service on Sunday morning on board the yacht, Rear-Admiral Sir Berkeley Milne reading the prayers and the band of the Royal Marine Artillery leading the singing. In the afternoon a visit was paid by the Queen and most of the Royal party to the Convalescent Home for Naval and Military Officers at Osborne.

### Assassination of the Russian Minister of the Interior.

The civilised world was startled on Friday last by the official announcement that M. de Plehve, the Russian Minister of the Interior, had been assassinated. M. de Plehve was driving to the Baltic Railway station in St. Petersburg on his way to see the Czar at Peterhoff. While he was passing through the Ismailovsky Prospect a man threw a bomb from a window in a restaurant. It exploded and blew the carriage to pieces, instantly killing the Minister and his coachman, whose bodies were terribly mangled. Many other persons in the vicinity were also badly injured. The assassin, who was badly hurt, was immediately arrested, and in handing a revolver to the police, he said that they might do what they liked with him. M. de Plehve was born in 1848, and his whole life had been spent in the service of the State. He first rose to prominence by the energy he displayed in conducting the investigation into the assassination of Alexander II. His predecessor was assassinated in 1902 On hearing of the tragedy, King Edward telegraphed his sympathy to the Czar, who sent a grateful reply. The funeral of the deceased minister took place on Sunday, the Czar being amongst the mourners present. In connection with the assassination about a thousand persons were arrested on Saturday. It was stated on Tuesday that the assassin was dead, but it appears that he is gradually recovering.

### The War in the Far East.

Statements made in both Houses of Parliament with regard to the questions at issue between the British and Russian Governments, portend a satisfactory settlement. The Japanese report that at the battle of Ta-shih-chiao the Russian casualties were about 2,000, those of the Japanese being about half that number. General Count Keller was among the Russian killed. Up to nightfall on July 24, the Japanese attacks had been repelled, but a night attack made by the right wing proved successful, and after some hours' stubborn fighting the Russians evacuated their lines and retreated. A telegram from Tokio says that after three days' desperate fighting the Japanese have captured an important defence of Port Arthur.

### Women at the Sanitary Congress.

At the Congress of the Sanitary Institute held in Glasgow, which closed on Saturday, the Duchess of Montrose presided over a Conference of Women on Hygiene. Papers were read on the need for training girls in the care of infants, the management of children, the physical training of women and girls, and the hygiene of school life. Mr. James Graham, inspector of schools for the West Riding County Council, also read a paper, illustrated with photographs, giving particulars of adjustable desks and seats, such as are seen in Continental schools, and which are suitable for children of tender years. He urged that a desk should be used which would readily accommodate itself to the children's bodies, and to the needs of adult students. Another notable contribution was by Miss Norah Vynne on "Woman's Home Work in Relation to Comfort, Character, and Health," who contended that home-work has many advantages and no disadvantages, except that the sweater can directly fatten himself on the individual home-workers; but, she added, it was obvious that the remedy for that was to attack the sweater and not the home-worker. Several ladies, who joined in the discussion, did not agree with her.

### Death of Mr. Frederick Goodall, R.A.

The death of Mr. Frederick Goodall, R.A., took place on Friday at Hampstead. Mr. Goodall, who was in his 82nd year, had been ill for some time. He was the son of the well-known engraver, Edward Goodall, and inherited the artistic vocation. When he was only 14 he won the "Isis" gold medal at the Society of Arts, and first exhibited a picture in 1839. At 31 he became an Associate of the Royal Academy, and 10 years later a Royal Academician. In 1857 he visited the East, and in consequence he painted a number of Eastern pictures. In fact, it was largely to his Biblical pictures that he owed his popularity. He also painted several excellent portraits, including one of Mr. Gladstone, and late in life he addressed himself to landscape, "The Thames from Windsor," being one of his successes. In 1902 he had to sell his beautiful house in Regent's Park, though at one time of his career the income derived from his pictures was £10,000 a year.

### Pneumatic Despatch Tubes.

A small plant with 850 feet of tubing has been laid down by the Batcheller Pneumatic Despatch Company at Ranelagh Lodge, Fulham, to demonstrate their system of conveying mails, parcels, and light luggage through large underground tubes by air-pressure. It is proposed to promote a Bill in Parliament next year to authorise a pneumatic express service of this kind in London, with at least 150 receiving and despatching stations. It is claimed for the Batcheller scheme that in operation it would not only give quicker delivery and cost less than any of the existing methods, because of the increased speed of transmission and the reduced number of handlings but would also take away one-sixth of all the vehicles now using the streets, and thus save scores of thousands of pounds yearly in road repairs. The demonstration now proceeding excites considerable interest, and seems to be altogether satisfactory.

### The August Bank Holiday.

Lovely weather prevailed all over the country on Bank Holiday, and in consequence several of the railway companies surpassed their previous records in holiday traffic. For instance, the Great Eastern Company conveyed on Monday from their London stations to places in the suburbs about 121,781 passengers, as compared with 107,383 on the corresponding day in August last year. The increase in the total of passengers taken by ordinary trains at Paddington between Saturday morning and Monday at noon was 2,250. The Brighton and South Coast Company despatched between Friday and Sunday from London 2,450 cycles; 77,000 persons went to Kew Gardens; 63,000 to Earl's Court, 60,000 to the Crystal Palace, and 25,000 to Highgate Woods. There was, on the other hand, a noticeable diminution of visitors at museums and indoor exhibitions.

### Notes and Queries.

**FOR REGULATIONS SEE PAGE 143.**

*Hospital Training.*

(134) Will you kindly tell me if it will be any advantage to enter my name on the books of the National Hospital? I do not propose entering until next year.—*I. M. F.*

Write to the Matron.

*By Letter.*

(135) A correspondent requests us to answer her question "by letter and not through your paper." She, however, encloses no fee. A very large number send stamped envelopes for private replies and also omit to enclose the fees of 2s. 6d. each. Much trouble and disappointment would be saved if they would read the rules.

*Secretary.*

(136) I am a rapid writer, a good typist, hold certificates. Am I competent for the post of secretary to a doctor?—*Edith.*

Yes; the difficulty is, of course, to obtain such a post. Write to the Secretary, the Central Bureau for the Employment of Women, 9 Southampton Street, High Holborn, W.C.

*Salisbury Diet.*

(137) Will you kindly tell me what is the "Salisbury Diet," and is there a book on the subject?—*E. J. B.*

See "What must I do to get Well?" Published by the Scientific Press, price 6s. 3d., post free.

*Dispensers.*

(138) Can you tell me how to set about qualifying myself as a dispenser? Will it be necessary to reside in London? What is the shortest time I could do it in, and what will it cost me?—*Nurse L.*

Write to the Secretary of the Pharmaceutical Society, 17 Bloomsbury Square, W.C., for particulars.

Can you tell me where a nurse can prepare for the position of lady dispenser?—*C. F.*

See reply to *Nurse L.*

I should be much obliged if you could tell me of any school or classes in or near Liverpool where I could learn dispensing?—*M. H.*

See reply to *Nurse L.*

*Agency.*

(139) Will you kindly tell me if there is a society in Glasgow or Edinburgh which would send backward and neglected children to a trained nurse in the country?—*A. B.*

Possibly the Secretary of the National Association for Promoting the Welfare of the Feeble-minded, 53 Victoria Street, London, S.W., might be able to help you.

*Cleaning Sponges.*

(140) Will you instruct me in a method of c'eaning sponges from grease and *débris* after breast and similar operations? I have tried common soda and ammonia, but find neither entirely satisfactory.—*Inquirer.*

Wash in warm water (100° F.). Then transfer to warm solution of washing soda (ʒi to Oj of water) for half an hour. Then rinse the sponges well in warm, sterilised water, and immerse them in a cold solution of sulphurous acid (1 in 5) for 12 hours to sterilise them. The sponges should then be well washed in warm, boiled water and kept in 1 in 20 carbolic acid until further use. Unless they have been conscientiously disinfected in this way, sponges should on no account be used a second time.

*Fever Training.*

(141) A would-be nurse wishes to know the names of Fever Hospitals in or near London.—*M. A. W.*

Apply to the Secretary of the Metropolitan Asylums Board, The Embankment, London, E.C.

**Important Nursing Text-books.**

"The Nursing Profession: How and Where to Train." 2s. net; 2s. 4d. post free.

"The Nurses' Dictionary." By Honnor Morten. 2s. post free.

"Nursing: a Complete Text-book of Medical, Surgical, and Monthly Nursing." By Percy Lewis, M.D. 3s. 6d., post free.

"Elementary Physiology for Nurses." By C. F. Marshall, M.D. 2s. post free.

"A Complete Handbook of Midwifery for Nurses." By J. K. Watson, M.D. 6s. net; 6s. 4d. post free.

The above works are obtainable of all booksellers, or direct from The Scientific Press, Limited, 28 & 29 Southampton Street, Strand, London, W.C.

### For Reading to the Sick.

### THE WORD OF GOD.

LORD, Thy word abideth,
And our footsteps guideth;
Who its truth believeth
Light and joy receiveth.

When our foes are near us,
Then Thy word doth cheer us;
Word of consolation,
Message of salvation.

When the storms are o'er us,
And dark clouds before us,
Then its light directeth,
And our way protecteth.

Word of mercy, giving
Succour to the living;
Word of life, supplying
Comfort to the dying.

*Henry Baker.*

In the sublime prayer of our Lord for His disciples, on the eve of His Passion, He boasted that He had given the word of God to men as a legacy and guide, as their treasure and food. "I have given them Thy word," He said; "sanctify them through Thy truth: Thy word is truth." A word is not a mere sound; it is the expression of an idea or fact conceived in one mind and flashed across and received by another. Thus the word of God is intimately connected with God Himself; nay, it is a part of God, if one may use such a phrase; it is an expression of the mind of God for our benefit, enlightenment and support, that we might accept and love it as our model and standard, as our guide, with a voice to warn and cheer us by "showing us the Father." "Thy word, O my God, is a lamp to my feet and a light to my path."

Jesus Christ brought the word of God to earth; it is good tidings of great joy to all people. Jesus Christ *is* the word, and *lived* it for 33 years, for He was always Himself, and then told His apostles to preach it throughout the world. And they "preached Jesus"; they preached "Jesus crucified," and the word of God has been sown broadcast. Wherever there is a tabernacle, there is Jesus of Bethlehem, of the House of Bread; wherever there is a true home, there is Jesus of Nazareth; wherever there is a sick-bed or a desolate heart, there is Jesus of Calvary.

So that "not by bread alone do we live, but by every word that proceedeth from the mouth of God." Yes, by every word; we cannot disjoint its sentences, but must take it as a whole, as the grand deposit of truth and divine concession of God to men, to be received with meekness, for it is able to save our souls. It answers every question and solves every problem of life; it lies within the grasp of the poor and unlettered, yet furnishes matter for a life's study to the learned; it warns the foolish, it encourages the weak, it proclaims God's judgments upon wilful sin, and discloses God's gift and rewards to men; it consoles the afflicted and fixes the mind on eternity. Like some bell from a church tower, it peals on amid the voices of the universe telling us of our true end and pointing out the means of getting there.—*Anon.*

